# Primary Lung Adenocarcinoma Manifesting as Bilateral Reticulonodular Infiltrates: A Case Report

**DOI:** 10.1155/crpu/6678388

**Published:** 2025-06-09

**Authors:** Geran Maule, Qusai Alqudah, Mohamed Ismail, Ryan Fox, Ajaypal Gill, Luis Javier

**Affiliations:** ^1^Department of Internal Medicine, University of Central Florida College of Medicine, Orlando, Florida, USA; ^2^Department of Graduate Medical Education, HCA Florida North Florida Hospital, Gainesville, Florida, USA

## Abstract

A 52-year-old female with a history of gastroesophageal reflux disease (GERD), hypertension (HTN), and supraventricular tachycardia (SVT) status postablation presented with progressive dyspnea and diffuse bilateral infiltrates on imaging. Symptoms began following exposure to a chicken farm, initially as a dry cough, evolving despite treatment with antibiotics, albuterol, and methylprednisolone. Emergency department CT imaging demonstrated bilateral linear, reticular, and nodular infiltrates. A negative infectious workup prompted bronchoscopy, confirming lung adenocarcinoma via immunohistochemical staining despite no significant smoking history, international travel, or other exposures. Brain MRI identified a solitary 7-mm enhancing lesion, guiding subsequent oncologic management. This case underscores the complexity of diagnosing atypical pulmonary symptoms and advocates for early bronchoscopic evaluation in suspected malignancies, particularly with pertinent family history.

## 1. Introduction

Lung adenocarcinoma is the most common subtype of non-small cell lung cancer (NSCLC) in the United States, characterized by its glandular differentiation and propensity for distant metastasis [1]. This malignancy accounts for approximately 40% of all lung cancers and typically arises from the peripheral lung tissue. While smoking remains a primary risk factor, adenocarcinoma is increasingly recognized in nonsmokers and individuals with diverse environmental exposures [1]. A reticulonodular interstitial pattern on a chest radiograph arises from either the overlapping of reticular shadows or the combination of reticular shadowing and pulmonary nodules. Few diseases are pathologically confirmed to exhibit this pattern, such as silicosis, pulmonary sarcoidosis, and lymphangitis carcinomatosis [2].

In clinical practice, the presentation of lung adenocarcinoma can vary widely, ranging from asymptomatic incidental findings to advanced stages with debilitating respiratory symptoms. Common initial symptoms include cough, dyspnea, and occasionally hemoptysis, reflecting the tumor's invasive nature within the lung parenchyma [3]. Imaging plays a pivotal role in the diagnostic pathway, with computed tomography (CT) scans often revealing characteristic features such as nodular opacities, ground-glass opacities, or consolidations. Following UK NICE guidelines, all patients with potentially curable lung cancer should undergo PET-CT for staging and treatment planning [4].

Herein, we present the case of a 52-year-old female who presented with worsening pulmonary symptoms and diffuse bilateral infiltrates on imaging. Despite initial considerations for infectious etiologies, further evaluation revealed lung adenocarcinoma, underscoring the diagnostic challenges and comprehensive approach required in managing atypical pulmonary presentations.

## 2. Case Presentation

The patient is a 52-year-old female with a significant past medical history of gastroesophageal reflux disease (GERD), hypertension (HTN), and supraventricular tachycardia (SVT) status postablation who was transferred to our facility due to progressively worsening shortness of breath and extensive diffuse bilateral infiltrates on Imaging.

The patient's symptoms began 2 months before her hospital presentation, following a visit to her family's chicken farm. She developed a dry cough and shortness of breath being progressively worsening despite receiving outpatient treatment with antibiotics, albuterol and methylprednisolone. Due to a significant exacerbation of her shortness of breath, she sought care at the emergency department (ED). A CT scan of her chest revealed extensive bilateral linear reticular and nodular infiltrates.

The patient denies ever smoking, excessive alcohol use, chronic steroid use, travel outside the country, incarceration, intravenous drug use, malignancy, history of tuberculosis (TB), chronic obstructive pulmonary disease (COPD), asthma, allergies, human immunodeficiency virus (HIV) infection, or exposure to pigeons or bats. Additionally, the patient noted a significant weight loss of approximately 32 kg over the past 10 months, which she attributes to maintaining a “healthy diet.” Notably, she has a significant family history of lung cancer, including her brother, who recently passed away, and her mother, who is also affected by the disease.

**Figure 1:**
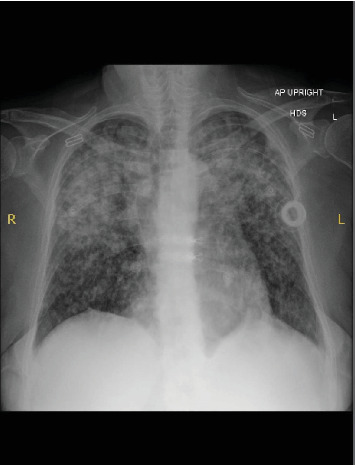
Anterior–posterior chest x-ray showing a bilateral ill-defined nodular pattern.

**Figure 2:**
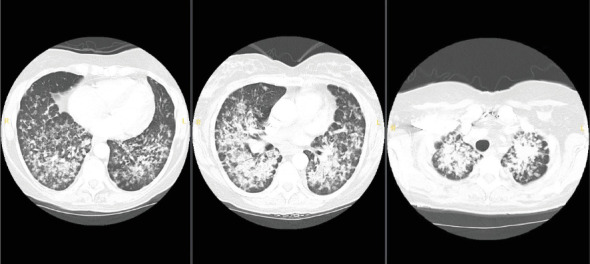
CT scans of the chest showing diffuse bilateral linear reticular and nodular infiltrates.

Upon arrival at our ED, the patient was hemodynamically stable with blood pressure (BP) of 136/78 mmHg, heart rate (HR) of 110 beats per minute, respiratory rate (RR) of 16 breaths per minute, and oxygen saturation (SpO2) of 98% on 4 L of nasal cannula (NC). The physical exam was notable for bilateral crackles, with no lymphadenopathy appreciated. Laboratory results were significant for a white blood cell (WBC) count of 15.1 × 10^3^* μ*L and lactic acid of 3.9 mmol/L. A chest X-ray (CXR) revealed an intermediate-sized ill-defined nodular pattern distributed throughout both lungs (Figures 1 and 2).

Given the suspicion of complicated pneumonia with potential etiologies including fungal infections and TB, isolation precautions were implemented. The patient was started on oral voriconazole pending further diagnostic information, as histoplasmosis infection was high on the differential diagnosis list. The workup included blood cultures, sputum cultures, acid-fast bacilli (AFB) staining, urine histoplasma antigen, galactomannan assay, beta-D-glucan assay, coccidioidomycosis serology, HIV serology, and cryptococcal antigen (crypto Ag) test in the blood. Chemical pneumonitis from environmental exposure was considered early in the differential; however, the lack of improvement with corticosteroids from her primary care provider and the imaging pattern made this less likely. Additional tests performed were COVID-19, influenza, urine *Streptococcus* antigen, and *Legionella* antigen tests.

Transbronchial biopsies were performed in the apical and anterior segments of the right upper lobe (RUL) of an area of nodular infiltrates. Also, endobronchial biopsies of nodular mucosa were performed in the left upper lobe. Bronchoalveolar lavage (BAL) was performed in the right upper RUL anterior segment and sent for cell count and differential, routine cytology, and bacterial/acid-fast bacteria/fungal analysis. The biopsies confirmed evidence of lung adenocarcinoma. Immunohistochemical (IHC) stains showed tumor cells positive for CK7, TTF-1, and napsin A, with patchy, weak nuclear reactivity for CDX2. CK20, GATA-3, and PAX-8 were negative. These morphologic and immunophenotypic findings were consistent with lung adenocarcinoma. Lymphangitic carcinomatosis was not reported in the histologic or IHC findings.

Brain MRI revealed a 7-mm contrast-enhancing lesion in the left frontal lobe, presumed to be metastatic, for which the patient underwent stereotactic radiotherapy. A few weeks after discharge, a positron emission tomography/computed tomography (PET/CT) scan demonstrated intensely fluorodeoxyglucose (FDG)-avid bilateral pulmonary nodular opacities, predominantly in the upper lobes, with a maximum standardized uptake value (SUV) of 10.1. Additional findings included FDG-avid mediastinal and hilar lymphadenopathy, bilateral adrenal metastases (SUV max 5.7 on the right and 5.5 on the left), and extensive hypermetabolic osseous lesions involving the spine, pelvis, proximal femurs, and humeral head—consistent with widespread skeletal metastases. Treatment for adenocarcinoma was initiated with carboplatin, pemetrexed, and pembrolizumab every 3 weeks for four cycles, followed by maintenance Pembrolizumab. After two cycles, the regimen was discontinued following the detection of an EGFR mutation via molecular oncology testing, and the patient was transitioned to targeted therapy with Osimertinib. A repeat PET/CT approximately 14 months later demonstrated interval improvement, with decreased FDG avidity in nodal and osseous lesions and relatively stable pulmonary findings, suggesting a partial therapeutic response.

## 3. Discussion

In our case, primary adenocarcinoma of the lung was initially mistaken for an interstitial lung disease. The patient, a young female never smoker, presented with radiological findings that were atypical for primary lung malignancy. Initial imaging suggested an interstitial or infectious process, leading to an initial diagnosis of pneumonia. However, despite appropriate antibiotic therapy, the patient showed no clinical improvement. Although the patient attributed her weight loss to dietary changes, the extent and timeline strongly suggested unintentional weight loss, raising strong concern for malignancy. Further investigations, including advanced imaging and a biopsy, revealed the presence of adenocarcinoma.

The diagnosis of lung adenocarcinoma is confirmed through histopathological examination of tissue obtained via bronchoscopy, transthoracic needle biopsy, or surgical resection. IHC stains, such as CK7, TTF-1, and napsin A, aid in identifying the tumor's origin and subtype, distinguishing it from other lung cancers and metastatic lesions [5]. Given its aggressive nature and potential for early metastasis, prompt diagnosis and initiation of treatment are crucial. Treatment modalities encompass surgical resection for localized disease, chemotherapy, targeted therapies directed at specific genetic mutations (e.g., EGFR and ALK), and immunotherapy [6]. Radiotherapy remains a key component of both curative and palliative treatment strategies in select cases, as outlined in the NICE guideline NG122 on lung cancer diagnosis and management [4].

A thorough patient history, including family history and environmental exposures, is invaluable in guiding the differential diagnosis. A detailed patient history can uncover risk factors that may not be immediately apparent, leading to more targeted diagnostic efforts. For example, the patient's exposure to chickens initially raised concerns about infectious etiologies like histoplasmosis, which are endemic to certain geographic regions and associated with avian environments. Additionally, her notable weight loss over the past 10 months was a significant clinical clue that warranted further exploration beyond infectious causes. The presence of a strong family history of lung cancer, including her brother's recent death and her mother's ongoing battle with the disease, leads to the consideration of a malignant process despite the patient's nonsmoking status.

On imaging, adenocarcinoma typically presents unilaterally as solid or ground-glass nodules or masses, which are easier to recognize as potential malignancies. However, it can also present less commonly as bilateral nodular, interstitial, or ground-glass opacities [7]. These more atypical presentations can be particularly challenging to diagnose, especially in patients without a smoking history. In such cases, the opacities may initially be misinterpreted as infectious or inflammatory conditions, leading to delays in the accurate diagnosis of malignancy. In our patient, the spread of tumor cells to the pulmonary lymphatic system or adjacent interstitium resulted in the thickening of the bronchovascular bundles and septae, leading to diffuse interstitial opacities. This presentation can easily be misinterpreted as ILD, especially in patients with no significant smoking history [8]. Fluorodeoxyglucose positron emission tomography/computed tomography (FDG-PET/CT) played a critical role in this case by identifying extensive metastatic disease and clarifying the malignant nature of radiologically ambiguous findings.

Existing clinical guidelines and protocols for evaluating patients with atypical pulmonary presentations should be reviewed and updated to reflect the complexities highlighted by cases like this. Current guidelines often prioritize common infectious or inflammatory conditions, potentially leading to delays in diagnosing less common but critical conditions such as lung cancer in nonsmokers. Clinicians must recognize when to deviate from standard guidelines, particularly when initial treatments fail to resolve symptoms or when atypical features are present. This includes protocols for early bronchoscopic evaluation and tissue biopsy—recognizing that transbronchial biopsies may be inconclusive and, in some cases, more invasive sampling such as surgical biopsy may be necessary. Training and continuing education for healthcare providers should emphasize the importance of maintaining a high index of suspicion for less common conditions and the judicious use of advanced diagnostic tools. This approach ensures timely and accurate diagnosis, ultimately improving patient outcomes.

## 4. Conclusion

In conclusion, this case highlights the importance of considering malignancy in the differential diagnosis of unexplained pulmonary symptoms, particularly in patients with significant family histories of cancer and imaging findings that may be ambiguous. Early bronchoscopic evaluation and tissue diagnosis are crucial in guiding appropriate oncologic management and improving patient outcomes. It also touches on the fact that physicians must remain flexible and adaptable when initial treatments are ineffective, allowing them to shift their approach and consider new differential diagnoses.

## Data Availability

No datasets were generated or analyzed during the current study.
